# Enzymatic debridement for the treatment of severely burned upper extremities – early single center experiences

**DOI:** 10.1186/s12895-016-0045-2

**Published:** 2016-06-24

**Authors:** Tomke Cordts, Johannes Horter, Julian Vogelpohl, Thomas Kremer, Ulrich Kneser, Jochen-Frederick Hernekamp

**Affiliations:** Department of Hand, Plastic and Reconstructive Surgery – Burn Center, BG Trauma Center Ludwigshafen, Ludwig-Guttmann-Strasse 13, 67071 Ludwigshafen, Germany

**Keywords:** Burn wound, Enzymatic debridement, Bromelain, Scarring, Skin grafting, Plexus catheter

## Abstract

**Background:**

Severe burns of hands and arms are complex and challenging injuries. The Standard of care (SOC) – necrosectomy with skin grafting – is often associated with poor functional or aesthetic outcome. Enzymatic debridement (ED) is considered one promising alternative but, until recently, results proved to be highly variable.

**Methods:**

Between 04/2014 and 04/2015, 16 patients with deep partial- to full-thickness burns of the upper extremities underwent enzymatic debridement (ED) in our Burn Center and were evaluated for extent of additional surgery, wound healing, pain management and functional parameters.

**Results:**

Following ED, no further surgical intervention was required in 53.8 % of the study population. In patients who required surgical treatment, the the skin-grafted area could be reduced by 37.0 % when compared to initial assessment. Time from injury to ED was 24.4 h and patients were able to start physical therapy after 2.0 days but suffered from prolonged wound closure (28.0 days). Regionally administered anesthesia proved to be superior to pain medication alone as pain levels and consumed morphine-equivalent were lower. Post-demission follow-up showed good functional results and pain levels with low scores in two self-report questionnaires (DASH, PRWE-G) but 3 patients reported increased susceptibility to shear stress. Based on these early experiences, we developed a 3-step algorithm for consecutive patients allowing appropriate and individualized treatment selection.

**Conclusions:**

We see a potential benefit for ED in the treatment of severely burned hands and forearms but further investigations and proper prospective, randomized controlled trials are needed to statistically support any outlined assumptions.

**Electronic supplementary material:**

The online version of this article (doi:10.1186/s12895-016-0045-2) contains supplementary material, which is available to authorized users.

## Background

Burns are common injuries associated with substantial morbidity and mortality. Over 2000 cases have been treated in German Burn Centers in 2013 [1]. Estimates for the frequency of hand involvement vary between 30–60 and 80 % of newly admitted patients [2, 3]. Standard of care (SOC) for deep partial- and full-thickness burns is necrosectomy and skin grafting but, especially when conducted on hands and forearms, often associated with poor aesthetic and functional outcome [4].

Initial assessment of both burn extent and depth can prove to be difficult and inadequate. Additionally, burn wounds can progress and change over time, leading to an unanticipated need for surgery [5]. In combined partial- and full-thickness wounds, conventional surgical intervention may cause unnecessary tissue loss, since vital tissue might be unnecessarily removed. This can be detrimental for functional outcomes, especially when distal extremities with a thin soft tissue envelope are treated. Fingers and hands have a complex anatomy, sparse tissue coverage and present vessels, nerves and tendons confined on a very small space [4]. Since SOC might lead to unsatisfying outcomes in some patients, new therapeutic options are still required. Enzymatic debridement (ED) is considered one promising alternative and has therefore been extensively studied since the Second World War. But, until recently, results proved to be highly variable [6].

NexoBrid^®^ (NXB) is an enzymatic debriding agent that was EMEA-approved in late 2012. Its active ingredient constitutes of a concentrate of proteolytic enzymes enriched in bromelain derived from the stem of the pineapple plant. It is indicated for enzymatic debridement in adults with deep partial- and full-thickness thermal burns [7]. Application is possible outside the operation theatre as long as sufficient analgesia is ensured during this otherwise painful debridement procedure.

Previous studies reported promising results. A large multi-center, randomized controlled clinical trial conducted on 182 patients between 2006 and 2009 postulated significant reductions in time from injury to complete debridement, need for surgery, autografting and area of burns excised when compared to SOC. In a subgroup of hand burns, additional benefit was seen as these patients exhibited earlier time to wound closure and better long-term results [8]. Similar findings had previously been reported in a retrospective data analysis of 69 hand burns [2]. Furthermore, the number of escharotomies could be reduced, since initial ED sufficiently decreased compartment or interstitial pressures. In both studies, no ED treated hand required escharotomy compared to about 10 % in SOC [2, 8]. However, although these results suggest a benefit of ED especially in hand burns, no prospective correlative studies have yet been conducted.

In April 2014, we started using NXB on qualifying burns of the upper extremities as a possible alternative to surgical necrosectomy and skin grafting. We hypothesized, that these patients would benefit from reduced need for surgery and absent morbidity often associated with SOC, e.g. functionally impairing tissue loss and subcutaneous tissue damage. We also evaluated the time to onset of physical therapy (PT), wound closure and early functional outcome. Additional emphasize was put on pain management accompanying ED when comparing local to regionally administered anesthetic measures.

With this article, we want to provide an overview of the problems, experiences and results gathered when using ED for deep burns of the hands and forearms.

## Methods

Between 04/2014 and 04/2015, all patients admitted to our Burn Intensive Care Unit (BICU) showing deep partial- to full-thickness upper extremity burns were treated by ED on not more than 15 % TBSA within the first 48 h after admission. Fingers and hands were prioritized even when injuries were exceeding proximally or included additional body regions.

**Fig. 1:**
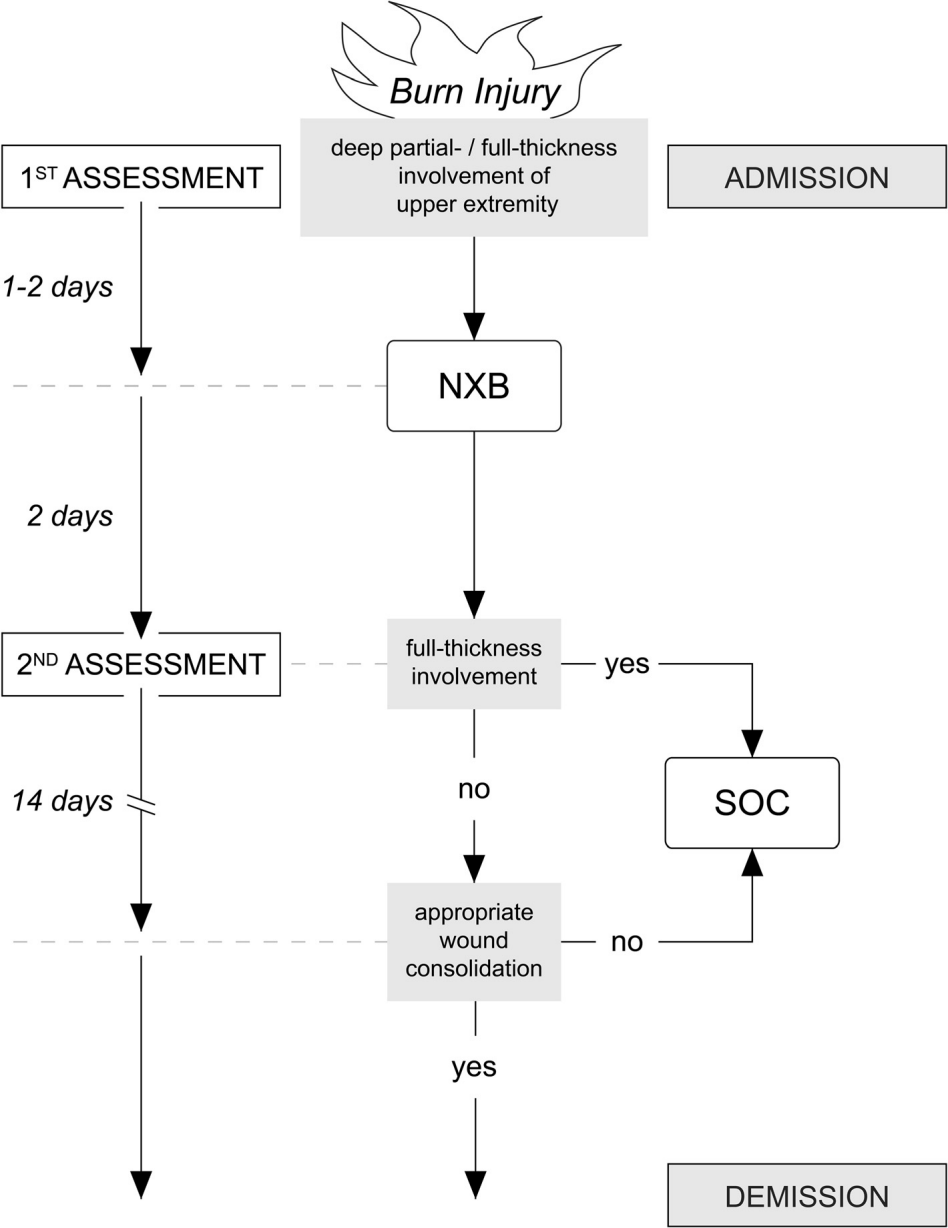
Ludwigshafen treatment algorithm for deep partial- to full-thickness burns involving the upper extremities

Upon arrival in the Burn Trauma Bay (BTB), standard admission protocol was performed which included complete undressing of the patient and initial debridement with opening of present blisters under antiseptic conditions. Standardized escharotomy by monopolar cautery was performed if injuries included circumferential burns. This was not regarded as exclusion criterion and ED was not performed as a replacement for surgical escharotomy. When wound bed was cleaned, a polihexanide gel was applied and sterile wound dressings were completed by multilayered greased gauzes, cotton and elastic bandages before admission to BICU. If burn depth on the upper extremities was categorized as potential deep partial- to full-thickness by the attending physician, dressings were opened and reassessed within 48 h (Fig. 1). If the patient qualified for ED, sufficient analgesia was ensured by either timely administration of p.o./i.v. pain medication or ultrasound-guided placement of brachial plexus nerve block by an anesthesiologist [9]. Additional pain medication was given if needed. Some patients received the application under anesthesia since severity of their total burn injuries had required intubation and ongoing sedation.

**Fig. 2:**
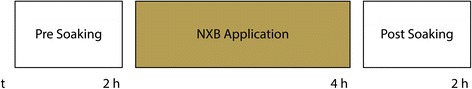
ED treatment phases

**Fig. 3:**
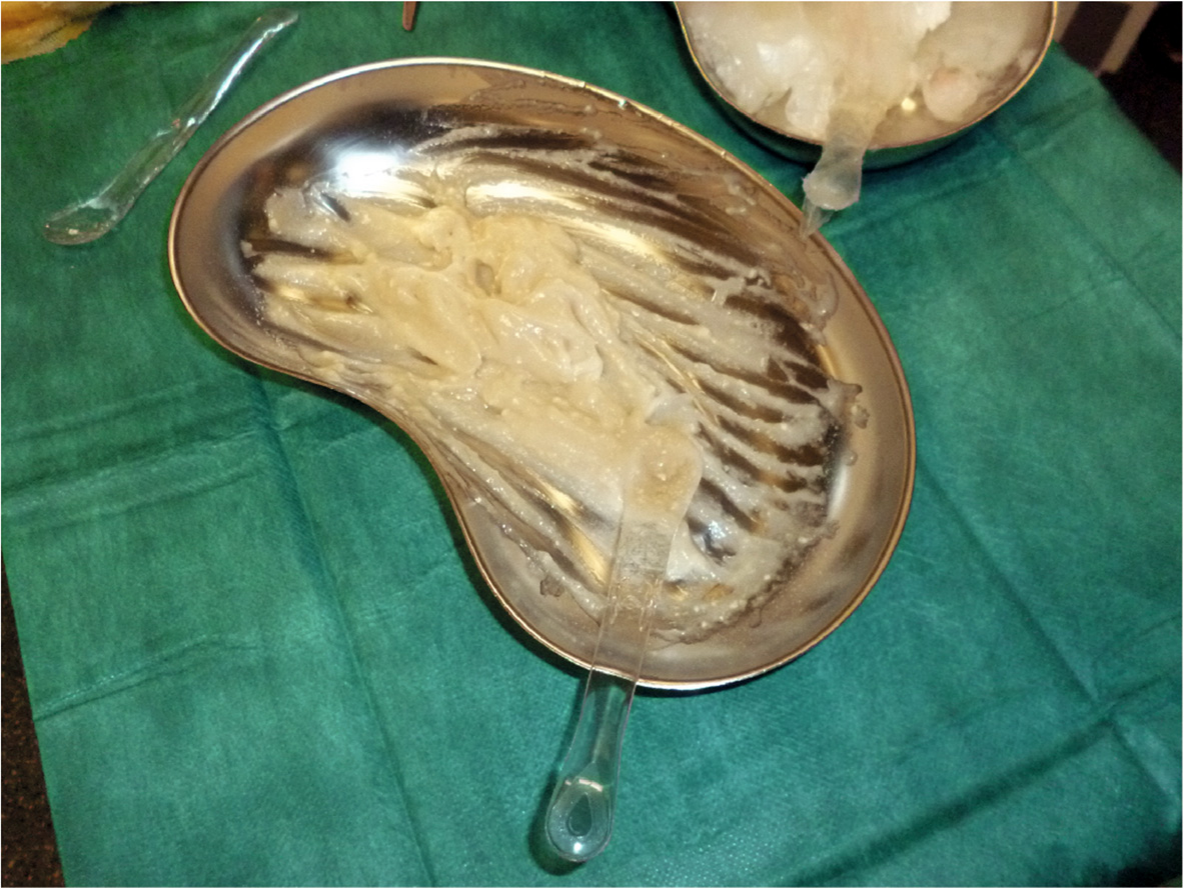
Active NXB agent after mixing powder ingredient and carrier gel

**Fig. 4:**
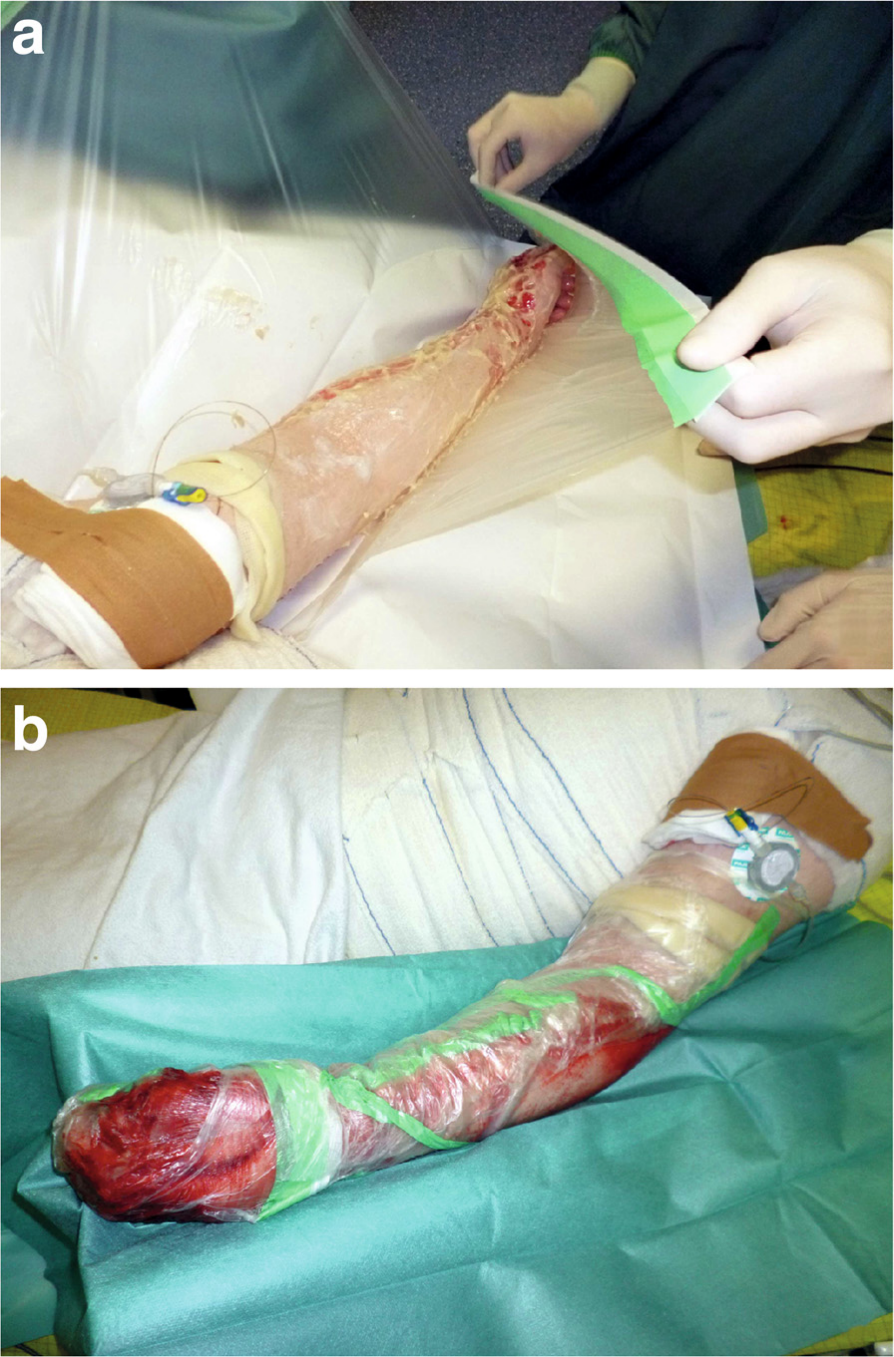
NXB application phase. **a** Forming of occlusive dressing by self-adhesing sheets. Plexus catheter already in place. **b** Completed dressing and active enzymatic reaction as indicated by bleeding

Wounds were covered in bandages rinsed in a 0.04 % polihexanide solution (Serasept^™^ 2, Serag-Wiessner, Naila, Germany) and soaked for 2 h before and after application of the actual debriding agent (Fig. 2). Preparation also included separation of the wound bed from intact skin by vaseline. Enzymatic reaction was then started by mixing the powder ingredient into the carrier gel, creating a golden viscous mass (Fig. 3) that was applied to the wound bed under sterile conditions (approx. 2 g/% TBSA). We then formed an occlusive dressing with two large plastic self-adhesing sheets (Fig. 4). Enzymatic debridement time was at least 4 h before dressings were opened and detritus was removed by sterile spatulas and rinsing in saline solution. The final step consisted of another 2 h soaking period before standardized greased gauze dressings were applied. In these first 16 cases, dressings were then changed every two days and reassessment was performed by an attending physician. To ensure proper and repeated wound bed evaluation, we abstained from using dermal substitutes or collagen matrices after the application of NXB.

In SOC, necrosectomy was conducted by Weck knife, Humby knife or Versajet™ (Smith & Nephew GmbH, Hamburg, Germany). We usually combined skin transplantation with spray application of fibrin sealant (ARTISS™, Baxter Deutschland GmbH, Unterschleißheim, Germany) to assure proper adhesion.

Throughout their stay on BICU, pain levels were monitored every hour, objectified by numeric rating scale (NRS) and patients received pain medication as required. Brachial plexus catheters were removed when analgesic effect decayed to insufficient levels or determined to be dispensable. Wound healing, signs of infection or other complications were assessed every two days by the attending physician when change of dressings took place. Following ED, physical therapy was started as soon as dressings would allow treatment by a professional therapist.

About 3 months after demission, patients were evaluated for remaining functional disabilities and pain levels using two German self-report questionnaires (Disabilities of the Shoulder, Arm and Hand – DASH; Patient-Related Wrist Evaluation Score – PRWE-G) as well as scar quality by a single physician (Vancouver Scar Scale – VSS).

## Results

Sixteen patients (11 male, 5 female) with burns of 20.1 (2–66) % TBSA were included in the study. On the upper extremities, 7.3 (2–15) % TBSA were classified as deep partial- to full-thickness and treated by ED 24.4 (5–47) hours after injury (Table 1). NXB proved to be very effective in achieving a complete initial debridement for all cases.

Individual data sets are provided as supplemental file, see Additional file 1: Raw data.

**Table 1:** Patient injury and treatment characteristics

Characteristic		
Epidemiology		
	Number of patients treated Mean age (SD), years Males, no. (%) Females, no. (%) Drop outs, no. (%)	16 47.8 ± 14.9 11 (68.8 %) 5 (31.2 %) 3 (18.8 %)
Injury and treatment		
	Mean (SD) % TBSA Mean (SD) % TBSA treated by ED Patients requiring skin grafting after ED, no. (%) Mean (SD) time to skin grafting after ED, days Mean (SD) % TBSA skin grafted after ED	20.1 ± 18.1 7.3 ± 4.1 6 (46.2 %) 16.3 ± 11.8 4.6 ± 4.1

Three patients deceased due to severity of accompanying injuries and therefore withdrew from follow-up. Out of the remaining 13, 7 patients completely avoided surgical intervention as necrosectomy by ED alone led to satisfactory wound healing. 6 required additional surgical debridement and skin grafting of 4.6 (0.5–12) % TBSA, equaling a 37.0 % reduction of transplanted skin area when compared to initial assessment. After ED patients were able to start PT after 2.0 (0–5) days but experienced prolonged wound closure with 28.0 (9–49) days on average.

**Fig. 5:**
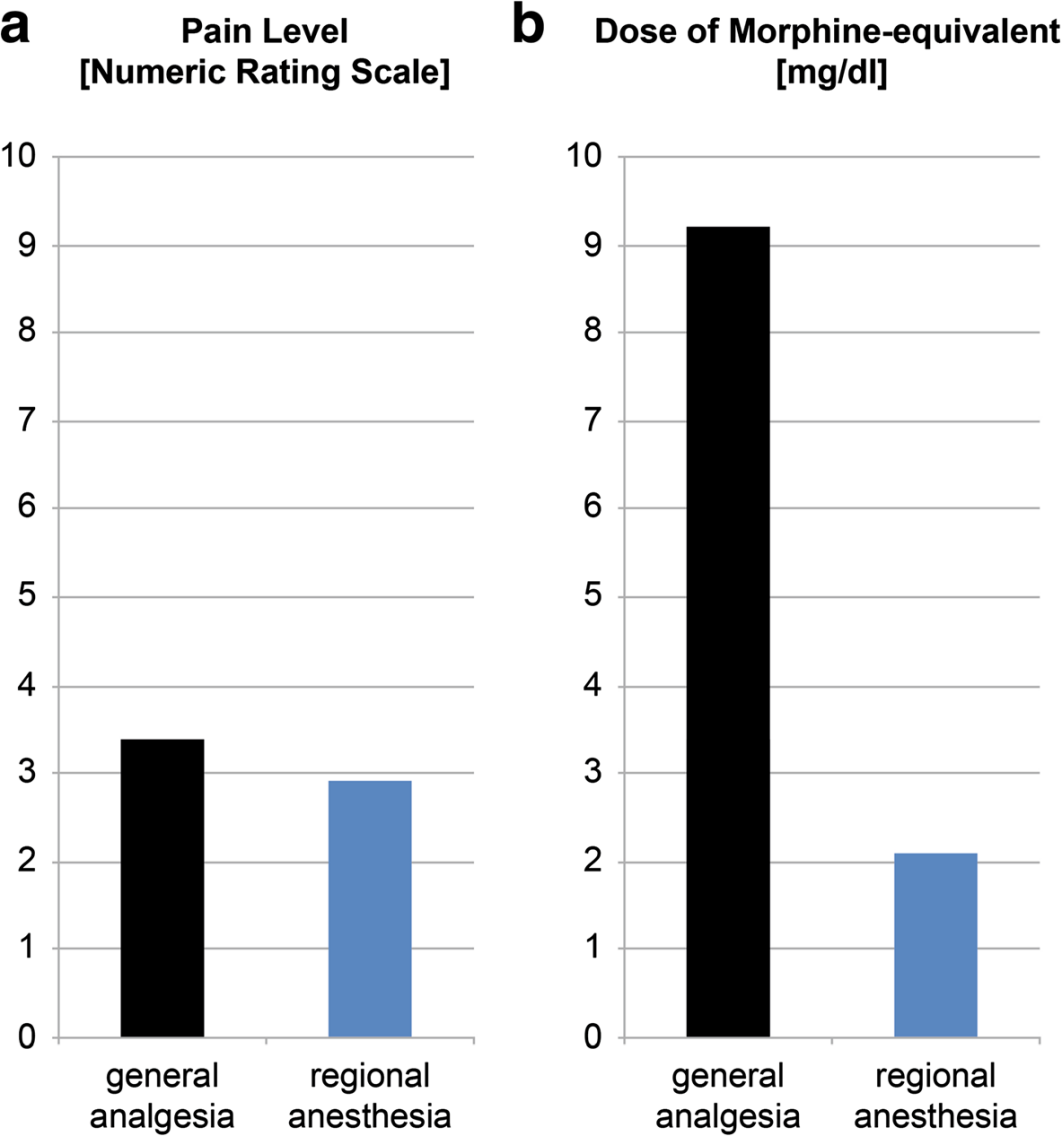
Comparison of general analgesia and regional anesthesia during ED. **a** Lower NRS scores in plexus catheter group. **b** Higher morphine-equivalent dose in general analgesia

Seven patients had been intubated on admission due to the severity of their injuries and were fully sedated during ED. According to the severity of the injury, patients underwent either p.o./i.v. pain medication (n = 3) or a brachial plexus nerve block was applied (n = 6). Regionally administered anesthesia proved to be superior to pain medication alone. Pain level, objectified by numeric rating scale (NRS), was 3.4 (1.1–5.2) in general analgesia and 2.9 (1.8–4.1) in plexus block group. This was accompanied by a 77.2 % reduction of morphine-equivalent consumed as general anesthesia group required 9.2 (0.0–17.2) mg/h and regional analgesia group required 2.1 (0.3–7.0) mg/h (Fig. 5).

No wound infections or ED-related side effects were seen. No blood transfusions were necessary.

**Fig. 6:**
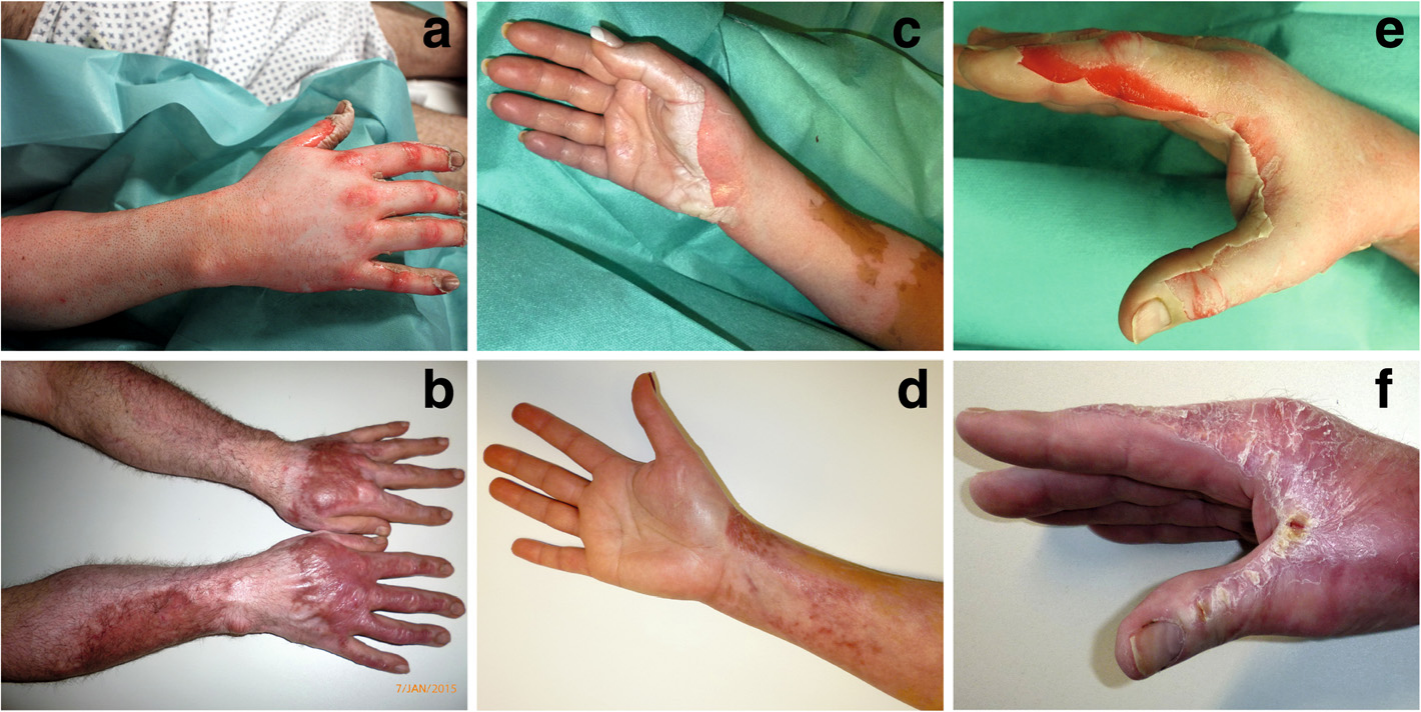
Three representative patients before and after ED. **a** and **b** Closed wounds 23 weeks after treatment. Note the intense vascularization in originally full-thickness affected areas. **c** and **d** Good results 16.5 weeks after ED. **e** and **f** Unstable scars with possibly sheer stressed induced interdigital lesioning still present after 18 weeks

Eight of 16 patients (50.0 %) were available for follow-up examination regarding remaining pain levels and functional outcomes. Results were within the lowest quarter of the scoring range with 23/100 (0–45) points on DASH and 22/100 (1–46) points on PRWE-G. Overall scar quality was assessed as 6/14 (4–8) points on VSS. All follow-up examinations were conducted by the same physician. 3 patients (37.5 %) complained about increased skin susceptibility of ED treated areas. Reports consisted of persisting lesioning (Fig. 6), partly spontaneous but mostly induced by minimal sheer stress or otherwise negligible, everyday trauma.

## Discussion

ED enabled patients to receive early initial debridement. With bedside application being practicable, patients would remain on ward and avoid cost-consuming surgical and anesthetic measures conducted in the OR. Also, in SOC, some days are usually allowed for wounds to completely demarcate the areas requiring necrosectomy and skin grafting, which further enhances time to debridement.

In our Burn Center, we were able to perform initial debridement 24.4 h after admission. ED phases itself will add up to at least 8 h of treatment (Fig. 2) and should therefore be started preferably in morning hours. Preceding plexus catheter placement further increases time by usually 1 or 2 h, rendering ED a long, laborsome procedure. We found this to be a limitation worth mentioning as ED requires a surgeon and nursing staff to be present at all times during the procedure.

ED reduced the % TBSA treated with surgical necrosectomy and skin grafting. Initial assessment of burn extent and depth is difficult and needs to be permanently scrutinized and reevaluated. Especially in the first days after injury, wounds can both progress or turn out to be overestimated by the admitting physician. The latter will result in unnecessary surgical interventions and morbidity, especially on delicate structures such as fingers and hands. In more than half of our collective initially classified as candidates for surgery, ED totally avoided necrosectomy and skin grafting. In the remaining patients, transplanted area was substantially smaller than initially anticipated, minimizing associated complications such as donor site morbidity, contractures and aesthetic disadvantages. Patients, however, exhibited prolonged wound healing and at least 14 days of close observation were allowed before additional debridement and skin grafting could be ruled out.

No differentiation was made between the treatment of deep partial- and full-thickness burns as NXB is approved for both. But experiences show that areas early identified as full-thickness often lacked any self-healing activity after ED. Furthermore, full-thickness wounds that successfully healed after ED were often observed with intense scar vascularization and discoloration as well as increased susceptibility to trauma (Fig. 6). Based on these findings, we changed our policy to earlier skin grafting of full-thickness areas after ED, actually combining SOC and ED (Fig. 1).

In these first 16 ED patients, no dermal substitutes or collagen matrices were used as application would have interfered with repeated wound assessment. However, products such as Matriderm™ (Dr. Suwelack Skin & Health Care AG, Billerbeck, Germany), Integra™ (Integra GmbH, Ratingen, Germany), Suprathel™(Polymedics Innovations GmbH, Denkendorf, Germany) and Biobrane™ (Smith & Nephew GmbH, Hamburg, Germany) are generally available and used in our Burn Center. Additional methods include allo- or xenografting and spray-on epidermal cells (Recell™, Avita Medical, Melbourn, United Kingdom). These products will presumably further facilitate burn wound consolidation but their exact effect on NXB treated areas is unknown and something we tend to elucidate in future investigations.

In our experience, ED does not necessarily require intubation and sedation, usually conducted in SOC. Pain is generally well tolerated by on ward anesthetic measures, with regionally administered analgesia being more effective when compared to p.o./i.v. medication alone. Ultrasound-guided placement of brachial plexus nerve blocks is safe and excludes morbidity possibly associated with intubation and sedation. These patients also exhibit decreased opioid doses essentially achieved by a continuously infused local anesthetic (mepivacaine or ropivacaine). Due to their additional anti-inflammatory properties, NSAIDs were given in both groups and were therefore not evaluated.

Grafted areas are immobilized for at least 5 days to ensure proper skin adhesion. This unfortunately excludes these regions from PT and subsequently leads to contractures, movement restrictions and often prolonged functional recovery. With ED, patients benefitted from early onset of PT measures, mostly on the first or second day after admission. 3-month follow-up also yielded good functional outcomes and pain levels.

## Conclusions

In conclusion, we see a potential benefit for ED in the treatment of deeply burned hands and arms but careful observation must be assured as full-thickness involvement might require treatment adjustments, possibly neglecting associated benefits. We found regional anesthesia by brachial plexus catheter being superior to pain medication alone but availability might be limited as placement usually requires a skilled anesthesiologist. Some patients complained of persistent lesioning and hypertrophic scarring which we attributed to our prolonged waiting for reepithelialization. Based on these findings, the following protocol is currently applied at our Burn Center (Fig. 1): All admitted patients with deep partial- to full-thickness burns of the upper extremities not meeting exclusion criteria (Table 2) will undergo ED within the first 48 h after trauma (1^st^ Assessment), preferably accompanied by regional anesthesia via brachial plexus blockage. Two days later, during dressing changes, the patient is again evaluated for areas of demarcated full-thickness involvement (2^nd^ Assessment), which would then be treated by additional necrosectomy and skin grafting. Absence of these would rule out SOC and allow wounds to consolidate for at least 2 more weeks before surgery is again taken into consideration.

**Table 2:** Applied NXB exclusion criteria

NXB contraindications as listed by manufacturer (see [7])	
Hypersensitivity to the active substance, to pineapples or papain or to any of the excipients	
Additional exclusion criteria applied in our Burn Center	
Pregnant and nursing women, Chemical or electrical burns, Age of burn injury >48 h	

We hereby report our experiences in using ED on 16 patients with deep partial- to full-thickness burns of the upper extremities. Although group size is small and obviously lacking appropriate controls, we feel that our observations will be of interest to any NXB using burn surgeon. Certain problems and obstacles had us adjust our initial treatment protocol but this current standard is solely based on expert opinion and clinical experience. Further investigations and prospective randomized controlled studies will be required to statistically support our conclusions.

## Abbreviations

BICU, burn intensive care unit; BTB, burn trauma bay; DASH, disabilities of the arm, shoulder and hand; ED, enzymatic debridement; NXB, NexoBrid^®^; PRWE-G, patient-related wrist evaluation score, German; PT, physical therapy; SOC, standard of care; TBSA, total body surface area

## Additional file

Additional file 1: Raw data. (XLSX 31 kb)
